# The Effects of Artificial vs. Natural Rearing on Growth Performance, Thyroid Hormone Levels, Locomotor Activity, Carcass Traits and Meat Quality Characteristics in Chios Lambs

**DOI:** 10.3390/ani15010054

**Published:** 2024-12-29

**Authors:** Panagiotis Simitzis, Georgia Alexopoulou, Eftychis Karampekos, Konstantina Linardopoulou, Anargyros Rigakis, Niki Stamelou, Michael Goliomytis, Iosif Bizelis, Ioannis Bossis

**Affiliations:** 1 Laboratory of Animal Breeding and Husbandry, Department of Animal Science, Agricultural University of Athens, 75 Iera Odos, 11855 Athens, Greece; stud212055@aua.gr (G.A.); karabekose@gmail.com (E.K.); ntina.lina@gmail.com (K.L.); rigakisanargyros@gmail.com (A.R.); nikistamelou@gmail.com (N.S.); mgolio@aua.gr (M.G.); jmpiz@aua.gr (I.B.); 2 Laboratory of Animal Husbandry, Department of Animal Production, Faculty of Agriculture, Forestry and Natural Environment, School of Agriculture, Aristotle University of Thessaloniki, 54124 Thessaloniki, Greece; bossisi@agro.auth.gr

**Keywords:** artificial rearing, dairy sheep, growth performance, behavior, thyroid hormones, carcass traits, meat quality, meat oxidative stability

## Abstract

Artificial rearing (AR) of lambs is nowadays a common practice in Mediterranean dairy sheep production systems to enhance the milk available for cheese or yoghurt manufacturing. The sufficient growth of lambs in an AR system is vital for the economic success of dairy sheep farms. However, AR is often associated with negative impacts on the performance and physiology of lambs. Greece is one of the major producers of ovine milk; nevertheless, data concerning the effects of artificial rearing in lambs of Greek autochthonous breeds are not available. Therefore, the present study aimed to examine the influence of artificial rearing on growth performance, thyroid hormone levels, locomotor activity, carcass traits and meat quality characteristics in lambs of the Chios breed, which is one of the most well-known Greek dairy sheep breeds. Twenty-one singleton male lambs were assigned into two feeding regimes; natural rearing NR (*n* = 11) and AR (*n* = 10). The lambs’ behavior was continuously videotaped until weaning, and their standing percentage was recorded as an activity index. Blood samples were collected from the jugular vein of lambs on days 3, 10, 17 and 40 after birth to assess thyroid hormone levels. The body weight of lambs was also recorded weekly. At the age of 45 days, lambs were fasted for 12 h, weighed and slaughtered. The weights of the carcass and internal organs were measured, while samples of the longissimus dorsi muscle were used for the determination of meat pH, color, water holding capacity, shear force and oxidative stability values. As indicated, body weight (kg) at birth was greater in NR vs. AR group and this difference was maintained till day 35 (*p* < 0.05), although body gain (kg) was generally not significantly different between NR and AR lambs, with the exception of the first week, when NR showed a greater value compared with the AR lambs (*p* < 0.001). On day 42, no significant differences between lamb groups for body weight were observed. Levels for triiodothyronine (T3), thyroxine (T4) and the free form of T3 (FT3) were greater, whereas the standing percentage was lower in NR compared with AR lambs (*p* < 0.05). The feeding regime of lambs did not affect carcass traits, internal organ and fat tissue weights, except for cold carcass yield which was greater in AR vs. NR lambs. No significant differences were observed between the two lamb groups in meat quality characteristics, such as pH, color, water holding capacity and shear force values, although MDA content was decreased in AR lambs indicating an improved oxidative stability. In conclusion, artificial rearing appears to be a feasible strategy for Chios lamb meat production, since it does not negatively influence carcass traits and meat quality characteristics, while a positive effect in meat oxidative stability is observed.

## Simple Summary

The increased global population and improved socioeconomic conditions have led to a dramatic surge in the demand for animal-derived products. Cheese and yoghurt originating from ewe’s milk are two of the most desirable dairy products around the world due to their exceptional nutritional and organoleptic properties. Artificial lamb rearing is a common practice in Mediterranean dairy sheep production systems to increase the quantity of marketable ovine milk. The results of the present study suggest that artificial rearing is a feasible strategy for lamb meat production in Greece since it has mild effects on endocrine and behavioral parameters, does not influence carcass traits and meat quality characteristics, and positively impacts meat oxidative stability.

## 1. Introduction

It is well-known that approximately 25% of the milk yield of each dairy ewe per lactation period is produced during the first 30 days after parturition [1]. It can be concluded that if lambs are left suckling till the 30th–45th day of their life, the farmer’s profit is significantly reduced, since lower quantities of marketable milk are produced [2]. Although the data are scarce, lambs should consume approximately 5.50 kg of milk to obtain 1 kg of body weight gain till the age of 28 days [3], indicating that this way of lamb rearing is uneconomical since meat prices are below 7 EUR/kg at the farmer’s level [4]. As a result, lambs are often separated from ewes at an early age and are artificially reared in Mediterranean dairy sheep production systems to increase the available milk quantity for cheese or yoghurt manufacture. However, specific care should be given for the adequate growth of lambs in an AR system, since lamb meat production is also essential for a profitable dairy sheep farm. According to previous studies, AR is often associated with a lower daily weight gain of lambs as a result of emotional (separation from the dam) and nutritional stress (from maternal milk to milk commercial substitute) [5,6].

In Mediterranean countries, most dairy sheep systems traditionally produce lambs slaughtered at 10–15 kg of live weight and aged up to 45 days old, particularly at Christmas or Easter, using indigenous breeds such as the Chios in Greece [7]. This type of lamb is a valuable product due to the high organoleptic qualities of the derived meat, such as its pale pink color, reduced amount of fat and subtle flavor [8]. It might thus be possible to obtain the maximum profit from the sale of lambs on the condition that carcass and meat traits are not negatively influenced by an artificial rearing feeding regime. However, the results are not consistent, since Napolitano et al. [9] found a greater carcass yield in AR compared with NR Comisana lambs, while the carcass weight was lower and the meat was darker and with a different fatty acid profile in AR compared to NR Barbaresca lambs, according to Lanza et al. [6].

Apart from direct observations associated with growth performance, alterations in blood thyroid hormone levels are an indirect index of adjustments in thyroid gland activity and circulating thyroid hormones reflect the nutritional and metabolic status of the animals [10]. Altered behavioral, endocrine and immune responses are also apparent in AR lambs. Specifically, an immunosuppressive effect is observed, since lambs fed with artificial milk show lower antibody production indicative of weaker humoral immune responses [5]. Adequate thyroid function is important for growth and development in neonates and it can be evaluated by the determination of thyroid hormone levels. In particular, triiodothyronine (T3) and thyroxine (T4) levels play a key role in the control of the metabolic rate of the developing lamb [11] and increased T3 levels are correlated with increased growth in males [12].

As one of the most producing and prolific breeds, Chios sheep are bred in Greece for their high milk yield, and it is hypothesized that lambs of this breed could be used in AR systems since they have the potential to cover the growing meat demands of the market and consumers. As far as the authors are aware, there is not a study providing data associated with AR in Chios lambs. Therefore, the aim of the present study was to evaluate the growth performance, behavioral activity, carcass traits and meat quality of artificially reared lambs and compare them with those of naturally reared ones.

## 2. Materials and Methods

### 2.1. Animals and Diets

Two-year-old ewes with similar body condition scores (2.5–3) which were at their second parity were randomly selected from the flock of the experimental farm of the Agricultural University of Athens and used in the present study. Within 2–4 h after parturition, which took place in November with a lambing interval of 2–3 days, the sex of lambs was confirmed and 21 singleton male Chios lambs were assigned into two groups: natural rearing (NR, *n* = 11) and artificial rearing (AR, *n* = 10) feeding regimes. The first group remained with their dams till weaning (45th day) and were provided with maternal milk as their sole feed, while the lambs of the latter group were removed within the first 24 h after consuming a sufficient quantity of colostrum and housed in a straw-bedded pen. Experimental groups were housed in two pens with outdoor access, each having the same direction and orientation and the same covered area (approximately 30 m^2^), while water was provided ad libitum. The conditions and facilities in each pen were checked twice per day during the experiment in order to ensure homogeneity among the treatments and avoid possible pen effects. AR lambs also had access to heat lamps during their first week of life.

Ewes of the NR group were fed a diet based on alfalfa hay and concentrates (Table S1) and the composition of their milk is presented in Figure S1. NR lambs spent the whole day with their dams; however, they did not have access to a maternal diet. On the other hand, AR lambs were fed with a milk replacer as their only feed (VIVALAC AC 60, Table 1). As reported, the milk replacer included 60% skim milk powder, whey powder, cereal byproducts, a mixture of vegetable oils, minerals and vitamins. AR lambs were offered the milk replacer at 37 °C, three times per day, from 4 collection buckets provided with latex teats (200 g of milk replacer in a total of 1 L) to avoid digestive disorders and according to the manufacturer’s guidelines.

### 2.2. Thyroid Hormones’ Determination

Blood samples were collected from the jugular vein of lambs at 10:00–11:00 a.m. in the morning on days 3, 10, 17 and 40 after parturition and allowed to coagulate spontaneously at room temperature. Serum samples were obtained by centrifugation at 5000× *g* for 15 min and stored at −20 °C until assayed for triiodothyronine (T3), thyroxine (T4) and their free forms (FT3 and FT4, respectively) by radioimmunoassay with commercial kits (KIP1631, KIP1641, KIPB1579 and KIPB1363, DiaSource ImmunoAssays S.A., Belgium for T3, T4, FT3 and FT4, respectively).

### 2.3. Locomotor Activity

The lambs’ behavior was constantly recorded till weaning, using 2 video cameras with infrared lighting (TX-1430OA, Turbo-X, Plaisio Computers, Metamorfosi Attikis, Greece). The number of lambs standing was noted through a camera that was placed in a fixed position in each pen, using time-lapse photography, every 10 min. It was considered that lambs were more active when standing compared with lying, so standing percentage was regarded as an index of activity. Data were then stored in a digital video recorder equipped with a hard disk (TX168, Telexper Inc., Union City, CA, USA).

### 2.4. Body Weight, Carcass Traits and Meat Quality Characteristics

The body weight of lambs was recorded on a weekly basis during the study. At the age of 45 days, lambs were fasted for 12 h (water was allowed), weighed and slaughtered. The weights of the heart, liver, spleen, kidneys, lungs and omental, intestinal, perirenal and pelvic fat were measured, since the development and metabolic activities of the visceral organs are strongly influenced by nutritional adequacy [13]. After carcass storage for 24 h at 4 °C, the *longissimus dorsi* muscle (6th–13th rib) was dissected and utilized for the analyses. The pH, color attributes, water holding capacity and tenderness (shear force values) were directly measured. The determination of lipid oxidation values was implemented on days 1, 4 and 8 and months 2 and 4 after storage at 4 °C and −20 °C, respectively.

#### 2.4.1. pH 24 and Color

A portable pH meter (HI 99163 model, Hanna Instruments, Cluj, Romania) was used by inserting the electrode into the *longissimus dorsi* muscle 24 h after slaughter. The standardization of the pH meter was carried out with buffers at pH 4.0 and 7.0 (Merck—Darmstadt, Germany) at ambient temperature. The part of the *longissimus dorsi* muscle between the 12th and 13th ribs was sliced across the fibers, left exposed to the air at room temperature for blooming for 30 min, and meat color was assessed in triplicate using a Miniscan XE (HunterLab, Reston, VA, USA) chromameter set on the L*, α*, b* system (CIE 1976, Commission International de l’ Eclairage). A white and a black tile were used for the calibration of the chromameter.

#### 2.4.2. Cooking Loss and Shear Force Value

A sample (80 ± 2 g) of the longissimus thoracis muscle from each lamb was weighed, placed in a plastic bag and cooked in a water bath at 75 °C for 35 min, left under tap water for 15 min and then left at room temperature. The sample was weighed again in order to calculate the percentage of cooking loss (%). Three sub samples with a cross section of 1 cm^2^ were then cut parallel to the muscle fibers and the shear force value of the longissimus thoracis muscle was measured using a Warner Bratzler (WB) shear blade fitted to a Zwick Testing Machine Model Z2.5/TN1S (Zwick GmbH and Co, Ulm, Germany). Peak force values in Newtons were recorded.

#### 2.4.3. Water Holding Capacity

The water holding capacity was determined according to the method suggested by Sierra [14]. Two muscle samples per lamb weighing about 5 g (P1) were put between two pieces of filter paper and pressed for 5 min, using a weight of 2.25 kg. The muscle samples were then removed and re-weighed (P2). The water holding capacity was determined as WHC (%) = (P1 − P2)/P1 × 100.

#### 2.4.4. Measurement of Adipocytes Number, Perirenal Adipose Tissue Cell Size and Total Lipids in Perirenal Tissue and Longissimus Thoracis Muscle

Directly after slaughter, a sample from the perirenal adipose tissue of each lamb was collected. A piece was placed in Krebs–Ringer bicarbonate buffer (pH: 7.4 at 37 °C) for the measurement of adipocyte size, whereas a second piece was immediately frozen and kept at −20 °C for the determination of adipose tissue fat content. The diameter of 100 adipocytes was assessed under a light microscope after separation by the collagenase digestion technique, as described by Rodbell [15]. The mean fat cell volume (V) of the 100 cells was calculated from the mean diameter (d) and the standard deviation (s), assuming a spherical shape of the fat cell, as follows: V = π(d^3^ + 3ds^2^)/6. The mean fat cell weight was obtained from the mean volume, assuming that the density of fat cells is that of triolein (0.915). The fat cell number per adipose tissue was obtained by dividing the total chemical fat of the tissue by the mean fat cell weight. The chemical fat content of the adipose tissue and longissimus thoracis muscle was determined according to the method first described by Folch et al. [16]. Tissue samples were homogenized with 2:1 chloroform–methanol mixture to a final dilution 20-fold the volume of the tissue sample. The crude extract was mixed with 20% of its volume of water and separated into two phases. The lower phase contained the tissue lipids.

#### 2.4.5. Measurement of Lipid Oxidation—MDA Assay

Lipid oxidation values were measured based on the malondialdehyde (MDA) formed during storage, a secondary lipid oxidation product formed by the hydrolysis of lipid hydroperoxides. In the present study, MDA levels in *longissimus dorsi* muscle samples were determined at 1, 4 and 8 days and 2 and 4 months after storage at 4 °C and −20 °C, respectively, using a selective third-order derivative spectrophotometric method previously developed by Botsoglou et al. [17]. Derivative versus conventional spectrophotometry was used since it provides improved sensitivity, specificity and reliability of measurements and eliminates potential interferences from other reactive compounds.

In brief, 2 g of each sample (in duplicate) were homogenized (Edmund Buehler 7400 Tuebingen/H04, Bodelshausen, Germany) in the presence of 8 mL aqueous trichloroacetic acid (TCA) (50 g/L) and 5 mL butylated hydroxytoluene (BHT) in hexane (8 g/L), and the mixture was centrifuged for 5 min at 5000× *g*. The top hexane layer was discarded and a 2.5 mL aliquot from the bottom layer was mixed with 1.5 mL aqueous 2-thiobarbituric acid (TBA) (8 g/L) to be further incubated at 70 °C for 30 min. Following incubation, the mixture was cooled under tap water and submitted to third-order derivative (3D) spectrophotometry (Hitachi U3010 Spectrophotometer, Tokyo, Japan) in the range of 500–550 nm. The concentration of MDA (ng/g wet tissue) in analyzed samples was calculated on the basis of the height of the third-order derivative peak at 521.5 nm by referring to the slope and intercept data of the computed least-squares fit of standard calibration curve prepared using 1,1,3,3-tetraethoxypropane (TEP), the malondialdehyde precursor.

### 2.5. Statistical Analysis

The adipocytes number, perirenal adipose tissue cell size and meat quality characteristics, such as pH24, color parameters (L*, α*, b*), intramuscular fat, cooking loss, water holding capacity and shear force value measurements for the longissimus thoracis muscle, were analyzed using a Mixed Model procedure which contained the fixed effect of nutritional treatment. Body weight, body weight gain, malondialdehyde (MDA) concentration, behavioral data (percentage of lambs standing) and thyroid hormones (T3, T4, FT3 and FT4) were analyzed using a Mixed Model appropriate for repeated measurements per subject, which included the effect of nutritional treatment as fixed effect. Lamb birth weight was fitted as a covariate in the analysis of body weight gain, because a significant difference was observed for birth weights. All model analyses were performed by Sas/Stat Version 9.1.3 [18].

## 3. Results

As indicated in Table 2, body weight (kg) was consistently higher in the naturally reared compared with the artificially reared lambs from day 0 till 35 (*p* < 0.05). On day 42, no significant difference was found between the two groups. On the other hand, weekly body gain (kg) was generally not significantly different between the naturally and artificially reared lambs with the exception of the first week, when ewe-reared lambs had a greater weight gain (more than twice) compared with the artificially reared lambs (*p* < 0.001).

As indicated in Table 3, T3, T4 and FT3 levels were steadily greater in naturally than artificially reared lambs (*p* < 0.05), apart from day 40, when no significant differences were shown. On the other hand, FT4 concentration was greater in AR vs. NR lambs on day 3, but similar values were observed between lamb groups on days 10, 17 and 40. Furthermore, serum T3, T4, FT3 and FT4 concentrations decreased with advancing age (*p* < 0.05) in both groups, with the exception of T3 in AR lambs.

**Figure 1:**
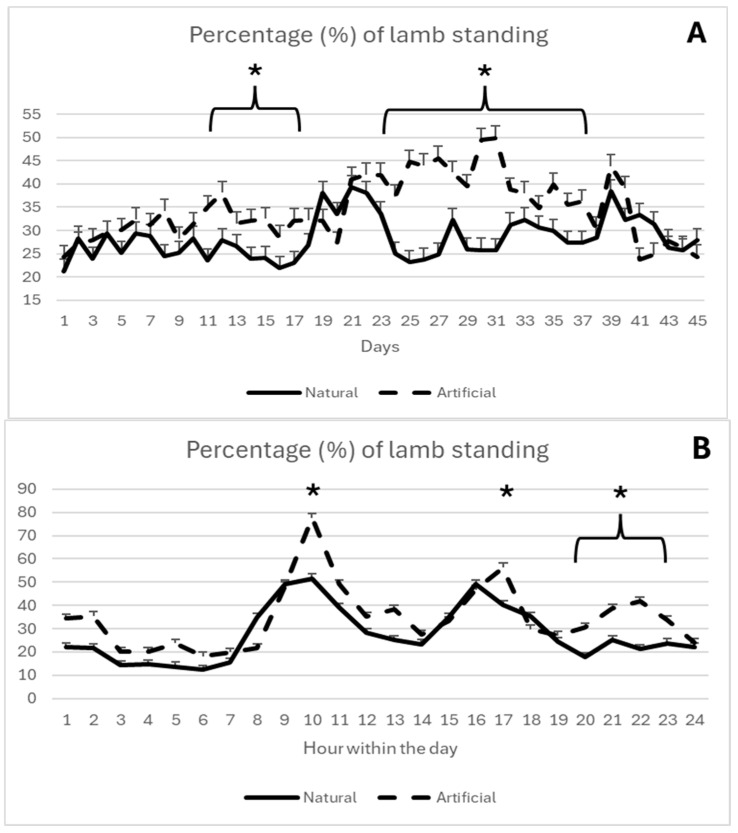
Percentage of lambs standing fed with natural or artificial milk within the experimental period (**A**) and within a day (**B**). * *p* < 0.05.

The mean percentage of standing lambs within the whole experimental period (0–45 days) was significantly higher in artificially fed compared with naturally suckled lambs (34.63 vs. 27.49 ± 0.004; *p* < 0.001). In particular, as shown in Figure 1A, there were two periods (11th–19th and 23rd–37th days) where the percentage of standing lambs and as a result the lamb activity was greater for artificially vs. naturally reared lambs (*p* < 0.05). Within the day, artificially reared lambs showed a higher standing percentage at 10 am, 5 pm and 8–11 pm.

As illustrated in Table 4, the feeding regime of lambs did not significantly affect carcass traits, internal organ weight or fat tissue weight (*p* > 0.05). The only significant difference was observed for cold carcass yield which was greater in artificially (57.23%) than naturally (55.31) reared lambs (*p* < 0.05). No significant differences were observed for meat quality characteristics, such as pH, color attributes, water holding capacity, cooking loss and shear force value, between the two lamb groups (Table 5; *p* > 0.05).

As reported in Table 6, MDA values were constantly lower in artificially vs. naturally reared lambs both after refrigerated (1, 4 and 8 days) and frozen (2 and 4 months) storage.

## 4. Discussion

As presented in Table 2, the significant differences for BW till the age of 35 days were not evident on day 42. This difference in body weight is partially the result of the initial difference in body weight at birth (day 0) rather than differences in body weight gain. However, the greater birth weight in NR compared with AR lambs was unexpected, since lambs were randomly selected from ewes of the same flock and assigned to the two experimental groups. Lamb birth weight was therefore fitted as a covariate in the lamb models used to analyze the remaining lamb growth traits. No mortality was observed in either lamb group.

Belanche et al. [19] reported that a higher neonatal growth was observed in naturally than artificially reared lambs during the first week of life, possibly attributable to the fact that the ewe’s maternal instinct helps lambs to suckle more efficiently. Oztabak and Ozpinar [20] and Napolitano et al. [21] showed that the average daily weight gain for ewe-reared lambs was significantly higher than that of artificially reared lambs, from birth up until 21 days. Belanche et al. [19] also found greater growth rates during the first month in naturally versus artificially reared lambs; however, the latter showed higher growth rates within the 31st-45th days, reaching similar weaning weights at 45 days of age, possibly due to their increased milk intake. De la Fuente et al. [22] reached similar conclusions; the daily gain between the 15th and 30th days was higher in animals reared artificially than in those fed by their mothers (310 vs. 245 g/day, *p* = 0.001). In general, the number of daily meals and their duration decreased while the quantity of milk consumed per meal and per day increased with age in artificially reared Romane lambs [23]. On the other hand, in naturally reared lambs, milk intake was decreased from the third week after birth which corresponds with the peak in the lactation curve [24]. In addition, there may be some compensatory growth effect in the lambs that were fed milk replacer, following the initial period of limited growth [25]. Napolitano et al. [9] and Sevi et al. [26] also found no differences in average growth rates between the artificially and naturally reared lambs. Damián et al. [27] and De la Fuente et al. [22] found no significant differences in body weight between naturally and artificially reared Polwarth and Colmenareña—Rubia del Molar male lambs, respectively. No significant effect was also observed in the body weight at 30 days and the postnatal growth rate from birth to 30 days between artificially and naturally reared lambs [1] and kids [28]. The contradictory results could be attributed to discrepancies in the composition and feeding regime of artificial milk (ad libitum or not), genotype (breed), supplementation of concentrates or forages or applied production system. Some parameters, such as sample size, environmental conditions or inherent variability in the growth or health status of lambs, could further affect the observed results.

As already pointed out, thyroid function affects growth and development in neonates, since T3 and T4 levels are strongly associated with the metabolic rate in the developing lamb [11], while increased T3 levels result in improved growth rates in males [12]. T3, T4 and FT3 levels were steadily greater in NR than AR lambs (Table 3). Firat et al. [29] reported similar findings, since they showed that lambs separated from their dams and fed artificial milk had lower serum T3 and T4 levels, possibly as an effect of neonatal hypotrophy or undernutrition [30]. Moreover, T3 and T4 levels decreased with advancing age, a finding that is in accordance with those of previous studies [29,31]. In a previous study, FT3 in neonatal lambs increased parallel to total T3, whereas the neonatal increase of free T4 (FT4) concentrations was greater and longer lasting than total T4 [30,32].

The locomotory activity of lambs was significantly greater in AR vs. NR lambs during the whole experimental period (0–45 days), with profound differences during two specific periods (days 11–19 and 23–37). Moreover, AR lambs showed an increased standing percentage at 10 am, 5 pm and 8–11 pm during the day. The first two hours (10 am and 5 pm) coincided with the hours of milk replacer provision. In contrast to our findings, Napolitano et al. [9] stated that an artificial rearing feeding regime did not have an effect on the duration of movement and number of bleats in lambs at the age of 42 days. However, Sevi et al. [33] reported marked withdrawal behavior and increased vocal responses in artificially reared lambs at the age of 10–20 days. The extended period for standing could be associated with impaired welfare and reflects the impact of psychological and physiological stressors induced by the early separation of the lamb and ewe [5,9].

Artificial rearing did not influence carcass traits, internal organ or fat tissue weight, with the exception of cold carcass yield that was greater in AR than NR lambs. Moreover, no significant differences were also observed for meat quality characteristics between the two lamb groups. Previous studies have reached similar conclusions, since although artificially reared lambs presented lower body weights than ewe-reared ones during the first month of their lives, no significant differences were shown for carcass traits (cold carcass weight, conformation score, fatness score) or meat quality parameters (pH, water holding capacity, color attributes) in North Country Mule lambs [34].

Belanche et al. [19] reported that although naturally reared lambs could have heavier final BWs, they tended to have a lower dressing percentage than artificially reared lambs and no significant difference was observed for carcass weight between the two treatments. Previous studies have also reported greater body weight gains for naturally versus artificially reared lambs [8,35]; however, cold carcass weight and meat quality characteristics, such as pH, cooking loss, shear force and color attributes, were similar [8]. No significant differences in carcass characteristics and perirenal fat levels between naturally and artificially reared lambs were also reported by De la Fuente et al. [22]. On the other hand, according to Chai et al. [36], artificially reared lambs had a higher average daily gain, slaughter weight, hot carcass weight and brighter meat color (lightness and yellowness) than ewe-reared Hu lambs. However, there were no differences in the values of water loss and drip loss between artificial and ewe-reared lambs, in accordance with the findings of Osorio et al. [37]. No significant differences in meat pH, redness, yellowness, chroma and hue angle between naturally and artificially reared lambs were also reported, while meat lightness and intramuscular fat was lower in AR vs. NR lambs [6]. To summarize, the results of the present study imply that any potential distress caused by artificial rearing did not have implications on the carcass or instrumental meat quality parameters.

Finally, MDA had lower values in AR than NR lambs after both refrigerated and frozen storage. According to previous studies, the proportion of polyunsaturated fatty acids is higher and that of saturated fatty acids lower in the meat derived from artificially than naturally reared lambs [6,9,36]. As can be concluded, the lower MDA values could be attributed to the protective role of the antioxidant (BHT) contained in the milk replacer used for the feeding of AR lambs, since polyunsaturated fatty acids are usually the category of acids that are oxidized.

## 5. Conclusions

The results of the present study show that an artificial rearing feeding regime did not negatively affect carcass traits and meat quality characteristics in Chios lambs, while a positive effect in meat oxidative stability and therefore shelf life was found. The observed differences in body weight at an early age could be attributed to the higher birth weight, but mostly to a brief low-consumption period during the transition to artificial milk replacer and they were not significant on day 42 and at slaughter. Differences in thyroid hormones and activity levels in artificially reared lambs possibly reflect mild alterations in endocrine and behavioral responses, respectively. To summarize, artificial rearing seems to be a feasible approach for Chios lamb meat production, since it does not affect carcass traits and meat quality characteristics, while a positive effect in meat oxidative stability is evident. However, the confirmation of the mechanisms involved warrants further research.

## Figures and Tables

**Table 1:** Chemical analysis of milk replacer.

Parameter	(%)
Crude protein	24.0
Crude fat	25.0
Crude fiber	0.10
Ash	7.00
Calcium	0.80
Sodium	0.50
Phoshorus	0.70
Vitamins and trace minerals	(mg/kg)
Vitamin A	15 *
Vitamin D3	2 *
Vitamin E	50
Vitamin C	75
Vitamin K3	4
Vitamin B1	16
Fe	35
Zn	65
Mn	55
Cu	3
I	0.4
Se	0.2
Antioxidant—BHT	30 mg/kg
Probiotic—Enterococcus faecum NCIMB 11181	1.3 × 10^9^ CFU/kg

* in kIU.

**Table 2:** Effect of natural or artificial milk feeding regime on body weight and body weight gain of Chios lambs.

Parameter	Sampling Day	Natural Rearing	Artificial Rearing	*p*-Value
Body weight (kg)	0	4.64 ± 0.18	3.95 ± 0.19	<0.05
	7	5.98 ± 0.18	4.61 ± 0.18	<0.001
	14	7.79 ± 0.29	6.26 ± 0.30	<0.01
	21	9.42 ± 0.40	7.63 ± 0.41	<0.01
	28	10.75 ± 0.51	8.89 ± 0.54	<0.05
	35	12.12 ± 0.63	10.19 ± 0.66	<0.05
	42	13.58 ± 0.74	11.76 ± 0.77	NS ^†^
Weekly body weight gain (kg)	0–7	1.34 ± 0.11	0.66 ± 0.12	<0.001
	8–14	1.81 ± 0.20	1.65 ± 0.21	NS
	15–21	1.62 ± 0.21	1.37 ± 0.22	NS
	22–28	1.33 ± 0.18	1.26 ± 0.19	NS
	29–35	1.37 ± 0.19	1.31 ± 0.20	NS
	36–42	1.46 ± 0.18	1.57 ± 0.19	NS
Daily body weight gain (kg)	0–42	0.21 ± 0.02	0.19 ± 0.02	NS

^†^ NS: Not Significant.

**Table 3:** Effect of natural or artificial milk feeding regime on the levels of thyroid hormones; T3 (ng/mL), T4 (ng/mL), FT3 (pmole/L) and FT4 (pmole/L).

Parameter *	Sampling Day	Natural Rearing	Artificial Rearing	*p*-Value
T3	3	4.41 ± 0.30 bA	2.27 ± 0.29 a	<0.001
	10	3.56 ± 0.29 bB	2.69 ± 0.29 a	<0.05
	17	3.83 ± 0.30 bAB	2.55 ± 0.29 a	<0.01
	40	3.18 ± 0.30 B	2.60 ± 0.29	NS ^†^
	*p*-value	<0.05	NS	
T4	3	125 ± 5.72 bA	92.0 ± 5.46 aA	<0.001
	10	99.5 ± 5.72 bB	83.8 ± 5.46 aAB	<0.001
	17	95.2 ± 5.72 bB	71.4 ± 5.46 aB	<0.01
	40	88.1 ± 5.72 B	85.4 ± 5.46 AB	NS
	*p*-value	<0.01	<0.01	
FT3	3	22.8 ± 1.12 bA	14.1 ± 1.07 aA	<0.001
	10	16.6 ± 1.07 bB	12.7 ± 1.07 aAC	<0.05
	17	13.6 ± 1.12 bC	10.5 ± 1.07 aBC	<0.05
	40	10.2 ± 1.12 D	9.81 ± 1.07 B	NS
	*p*-value	<0.05	<0.05	
FT4	3	26.5 ± 1.15 aA	32.6 ± 1.20 bA	<0.001
	10	26.6 ± 1.15 A	28.8 ± 1.15 B	NS
	17	22.9 ± 1.15 B	25.9 ± 1.20 C	NS
	40	22.8 ± 1.15 B	24.5 ± 1.20 C	NS
	*p*-value	<0.01	<0.05	

* Triiodothyronine (T3), thyroxine (T4) and their free forms (FT3 and FT4, respectively); ^†^ NS: Not Significant; ^a, b^ Values with different superscripts within a row and parameter are significantly different. ^A, B, C, D^ Values with different superscripts within a column and parameter are significantly different.

**Table 4:** Final body weight (FBW), hot–cold carcass weight (kg), carcass yield (%) and internal organ weight of lambs, as influenced by artificial rearing.

Parameter	Treatment		
	Natural Rearing	Artificial Rearing	*p*-Value
Final BW (FBW) at 45 days (kg)	13.13 ± 0.74	12.15 ± 0.78	0.373
Hot carcass weight (kg)	7.47 ± 0.46	7.09 ± 0.48	0.580
Cold carcass weight (kg)	7.31 ± 0.46	6.95 ± 0.48	0.598
Cold carcass yield (%)	55.31 ± 0.58 a	57.23 ± 0.61 b	0.034
Liver (g)	244.55 ± 14.02	244.00 ± 14.70	0.979
Heart (g)	74.36 ± 4.71	74.34 ± 4.93	0.997
Kidneys (g)	63.89 ± 3.54	66.32 ± 3.71	0.641
Lungs (g)	319.55 ± 22.11	334.50 ± 23.19	0.646
Spleen (g)	27.92 ± 1.89	28.82 ± 1.99	0.746
Adipocytes number (/g perirenal adipose tissue)	1180 ± 268	1064 ± 281	0.769
Perirenal adipose tissue cell diameter (μm)	60.21 ± 5.18	44.98 ± 5.43	0.057
Perirenal + pelvic fat (g)	51.00 ± 10.57	28.17 ± 11.08	0.152
Intestinal fat (g)	102.01 ± 8.55	82.49 ± 8.96	0.132
Omental fat (g)	54.98 ± 10.23	34.37 ± 10.73	0.181

a,b rates in a row with different superscripts are significantly different.

**Table 5:** Meat quality characteristics (pH, color, intramuscular fat, water holding capacity, cook loss and shear force value) of lambs as they are influenced by artificial rearing.

Parameter	Treatment		
	Natural Rearing	Artificial Rearing	*p*-Value
pH (24 h)	5.73 ± 0.03	5.66 ± 0.03	0.103
L*	45.73 ± 0.92	47.10 ± 0.97	0.316
Color a*	9.65 ± 0.27	9.55 ± 0.28	0.783
b*	12.10 ± 0.35	12.75 ± 0.36	0.211
Intramuscular fat (%)	1.54 ± 0.08	1.54 ± 0.09	0.991
Water holding capacity (%)	7.62 ± 0.66	6.59 ± 0.69	0.296
Cooking loss (%)	18.05 ± 1.00	19.84 ± 1.05	0.232
Shear force (N)	34.08 ± 1.26	32.45 ± 1.32	0.383

**Table 6:** Effect of refrigerated (at 4 °C) and long-term frozen (at −20 °C) storage on lipid oxidation of raw lamb longissimus thoracis muscle as affected by natural and artificial rearing (higher levels of MDA indicate higher rates of lipid oxidation).

	MDA (ng/g)	Treatment		
		Natural Rearing	Artificial Rearing	*p*-Value
Storage period (days, at 4 °C)	1	138.07 ± 22.69 a	58.08 ± 23.80 b	0.025
	4	585.63 ± 59.36 a	267.28 ± 62.26 b	0.002
	8	780.41 ± 102.15 a	506.60 ± 107.14 a	0.080
Storage period (months, at −20 °C)	2	389.20 ± 34.47 a	174.85 ± 35.15 b	0.001
	4	621.39 ± 96.16 a	318.46 ± 100.86 b	0.043

a,b rates in a row with different superscripts are significantly different.

## Data Availability

The data presented in this study are available upon request from the corresponding author.

## Supplementary Materials

The following supporting information can be downloaded at: https://www.mdpi.com/article/10.3390/ani15010054/s1, Table S1: Composition and analysis of dairy ewes’ diet; Figure S1: Milk fat, protein and lactose in NR ewes (5th–45th day of lactation).
