# Supplementary material for: The Effects of Artificial vs. Natural Rearing on Growth Performance, Thyroid Hormone Levels, Locomotor Activity, Carcass Traits and Meat Quality Characteristics in Chios Lambs
Source: Animals (Basel). 2024 Dec 29;15(1):54. doi: 10.3390/ani15010054 (PMC11718947; doi:10.3390/ani15010054)
Supplement: Supplementary file 1 [file animals-15-00054-s001.zip › animals-3379902-supplementary.pdf]

animals

Supplementary Materials

The effects of artificial vs. natural rearing on growth performance, thyroid hormone levels, locomotor activity, carcass traits and meat quality characteristics in Chios lambs

Table S1. Composition and analysis of dairy ewes' diet.

Ingredients (%)		
Corn	23.4	
Wheat	17.5	
Barley	17.5	
Soybean meal (44%)	18.25	
Sunflower meal (28%)	5.0	
Wheat bran	15.0	
Sodium chloride (NaCl)	1.0	
Limestone	1.85	
Monocalcium phosphate	0.4	
Vitamins and trace elements premix*	0.1	
Analysis	*Concentrates*	*Alfalfa hay*
Dry matter (%)	86.0	93.5
Crude protein (%)	17.0	10.2
Crude fibre (%)	6.0	34.2
Ash (%)	6.5	7.4
Fat (%)	2.1	2.3

*Premix contained per kg: 150mg Mg, 35mg Mn, 50mg Fe, 60mg Zn, 0.8mg Se, 0.75mg Co, 1.25mg I, 200mg Mo, 15 kIU vitamin A, 2 kIU vitamin D3, 25mg vitamin E (kIU: 1000 international units).

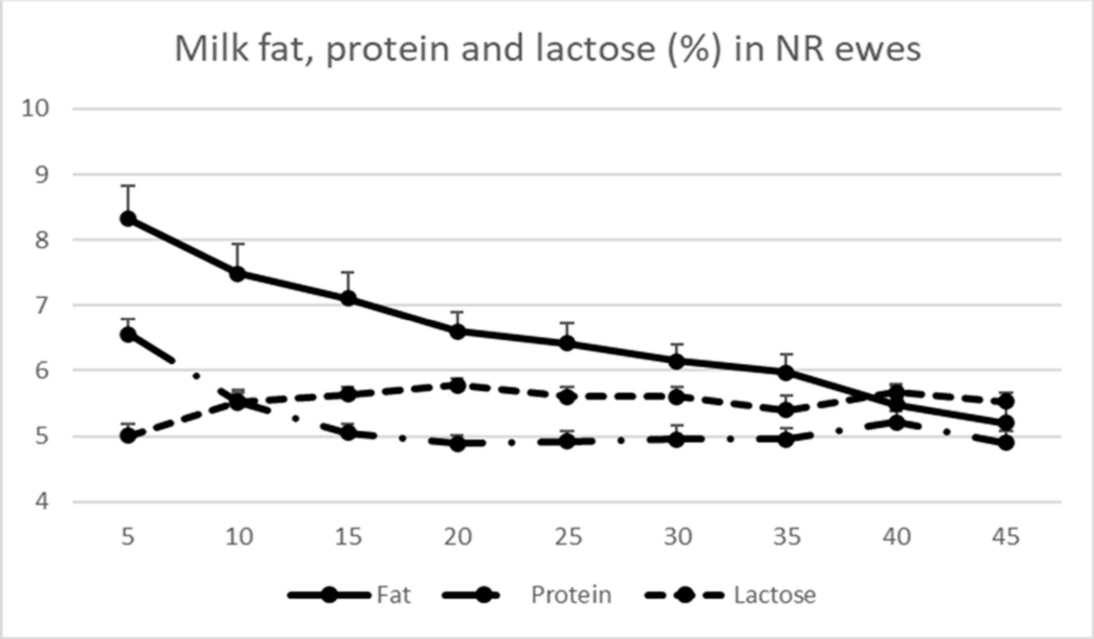

Figure S1 Milk fat, protein and lactose in NR ewes (5th-45th day of lactation)

Milk samples were analyzed for fat, protein and lactose using a Milkoscan 133 (Foss Electric, Hillerød, Denmark) calibrated for sheep milk according to the Mojonnier method for fat, the Kjeldahl method for protein and the polarimetric method for lactose on days 5, 10, 15, 20, 25, 30, 35, 40 and 45 of lactation.
